# Real‐world survival of patients with advanced BRAF V600 mutated melanoma treated with front‐line BRAF/MEK inhibitors, anti‐PD‐1 antibodies, or nivolumab/ipilimumab

**DOI:** 10.1002/cam4.2625

**Published:** 2019-11-02

**Authors:** Justin C. Moser, Danli Chen, Siwen Hu‐Lieskovan, Kenneth F. Grossmann, Shiven Patel, Sarah V. Colonna, Jian Ying, John R. Hyngstrom

**Affiliations:** ^1^ HonorHealth Research Institute Scottsdale AZ USA; ^2^ Division of Public Health, Study Design and Biostatistics Center Department of Family Medicine Huntsman Cancer Institute University of Utah Salt Lake City UT USA; ^3^ Division of Medical Oncology Department of Internal Medicine Huntsman Cancer Institute University of Utah Salt Lake City UT USA; ^4^ Surgical Oncology Department of Surgery Huntsman Cancer Institute University of Utah Salt Lake City UT USA

**Keywords:** anti‐PD‐1 antibodies, BRAF, dabrafenib, melanoma, nivolumab/ipilimumab, pembrolizumab, trametinib

## Abstract

**Background:**

The optimal treatment sequence for patients with advanced BRAF V600 mutant melanoma is unknown. BRAF/MEK inhibition (BRAF/MEKi), single agent anti‐PD‐1 (aPD‐1) antibodies and combination immune checkpoint inhibition with nivolumab and ipilimumab (niv/ipi) are all approved; however, they have not been prospectively compared. Therefore, we sought to compare overall survival of patients with advanced BRAF mutant melanoma treated with either front‐line BRAF/MEKi, aPD‐1, or niv/ipi.

**Methods:**

Patients with advanced BRAF mutant melanoma who had received BRAF/MEKi, niv/ipi, or aPD‐1 in the front‐line setting were identified from a nationwide database comprising de‐identified patient‐level structured and unstructured data derived from electronic health records. Survival was compared using Kaplan‐Meier curves and log‐rank analysis. Univariate and multivariate Cox regression models were used to measure the effect of front‐line treatment, age (>64 or not), LDH (elevated or not), and Eastern Cooperative Oncology Group (ECOG) performance status (>1 or not) on survival.

**Results:**

Five hundred and sixty seven patients with advanced disease and treated with front‐line aPD‐1 (n = 162), BRAF/MEKi (n = 297) or niv/ipi (n = 108) were identified. With a median follow‐up of 22.4 months, median overall survival (OS) for patients treated with front‐line niv/ipi was not reached (NR) while median OS for patients treated with aPD‐1 or BRAF/MEKi was 39.5 months and 13.2 months, respectively. Front‐line treatment with PD‐1 and niv/ipi were associated with statistically longer survival than BRAF/MEKi in multivariate analyses.

**Conclusions:**

In our real‐world retrospective analysis, patients with advanced BRAF mutant melanoma treated with front‐line niv/ipi or aPD‐1 had longer survival compared to those treated with front‐line BRAF/MEKi.

## Graphical abstract

Real‐world overall survival of patients with advanced BRAF mutant melanoma treated with front‐line BRAF/MEK inhibitors, anti‐PD‐1 antibodies, or nivolumab/ipilimumab.

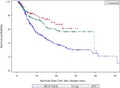

## 1 BACKGROUND

Roughly half of the cutaneous melanomas have been shown to harbor a BRAF V600 mutation.^1^ For patients with advanced melanoma whose cancer harbors a BRAF V600E/K (BRAF V600) mutation, the optimal front‐line treatment is unknown. Three different combinations of BRAF/MEK inhibitors (BRAF/MEKi) have been shown to be effective and are approved for use in patients with BRAF mutated melanoma.^2^, ^3^, ^4^ On the other hand, immune checkpoint inhibitors (ICI) are FDA‐approved and effective for patients whose melanoma harbors a BRAF mutation. Therefore, it is unclear whether targeted therapy with BRAF/MEKi or immunotherapy should be given in the front‐line setting and whether the sequence of these treatments impacts patient long‐term survival.

Cross trial comparisons suggest that initial response rates are higher for BRAF/MEKi compared to single agent anti‐PD‐1 antibodies (aPD‐1) and are similar to those for combined checkpoint inhibition with nivolumab and ipilimumab (niv/ipi). However, progression free survival (PFS) at 3 years appears to be lower for patients treated with BRAF/MEKi (roughly 20%) as compared to those treated with single agent aPD‐1 (roughly 30%) or niv/ipi (roughly 40%).^5^, ^6^ Additionally, retrospective studies have suggested cross resistance to ICI after progression on BRAF/MEKi.^7^ In this multicenter retrospective review, the median PFS for patients treated with front‐line aPD‐1 therapy was 10.8 months. However, for those who received aPD‐1 antibody after previously progressing on BRAF/MEKi, median PFS was only 2.8 months. Given the unclear optimal front‐line treatment for patients with advanced BRAF V600 mutated melanoma, we retrospectively compared the overall survival of these patients with front‐line aPD‐1, niv/ipi, or BRAF/MEKi.

## 2 METHODS

The Flatiron Health database, a longitudinal, demographically and geographically diverse database derived from de‐identified electronic health record (EHR) data, was reviewed for patients with advanced melanoma. The database includes data from over 280 cancer clinics (~800 sites of care) representing more than 2.1 million US cancer patients available for analysis. The patient‐level data in the EHRs include structured and unstructured variables curated via technology‐enabled abstraction. Research with the database was approved by the Copernicus Group Institutional Review Board (IRB) and received exemption from the University of Utah IRB.

Patients with advanced, metastatic, or unresectable, BRAF mutant melanoma who received treatment with front‐line aPD‐1, BRAF/MEKi, or niv/ipi were identified. Patients with incomplete clinical data or insufficient follow‐up (less than 30 days) from initiation of front‐line therapy were excluded. Overall survival (OS) from the initiation of front‐line therapy was compared among the three groups using Kaplan‐Meier curves and log‐rank tests. Known prognostic markers for melanoma including age >64 years, elevated (greater than upper limit of normal for the individual assay performed) pretreatment Lactate Dehydrogenase (LDH, obtained within 30 days of starting treatment), and elevated pretreatment performance status (PS) (Eastern Cooperative Oncology Group [ECOG] 2 or greater, obtained within 30 days of starting treatment) were also analyzed for their association with OS using univariate models. Multivariable Cox regression analysis was performed to compare the effect of the three treatments on survival from the initiation of front‐line therapy adjusted by age, ECOG, and LDH. Missing values of ECOG and LDH were categorized as one category so that observations with missing values could still be used in multivariable analysis.

Time to next‐line therapy or death (TTNTD) was selected as a surrogate for progression free survival to analyze treatment effect. TTNTD was calculated in a hierarchical matter where for patients who received second‐line systemic therapy, TTNTD was calculated from the initiation of front‐line therapy to the initiation date of second‐line systemic therapy. For patients who died without receiving second‐line systemic therapy, TTNTD was calculated from the initiation of front‐line therapy to the date of death. Patients who were alive without receiving second‐line systemic therapy at the time of analysis were censored at last known follow‐up. TTNTD was compared among the different treatment groups and prognostic markers in univariate and multivariate analyses, using the same statistical techniques for OS. Overall survival was based on EHR documentation plus linkage to external data sources as previously described.^8^

In order to assess if the difference in OS and TTNTD noted for patients treated with ICI as compared to BRAF/MEKi was due a potential bias of the broad inclusion criteria for patients within the dataset, OS and TTNTD were compared for a more restrictive set of patients. For this analysis, only patients who received the opposite or no therapy in the second‐line setting were included. Patients who received no second‐line therapy were included in the analysis in order to respect the proportion of long‐term responders for each treatment, and as to not introduce an immortal time bias into the analysis. This analysis was performed for Niv/ipi compared to BRAF/MEKi, and aPD‐1 and BRAF/MEKi separately. Hazard ratios for OS and TTNTD were calculated in a similar fashion as above.

## 3 RESULTS

Seven thousand six hundred and fifty patients with melanoma were identified from the Flatiron Health database. Of these, 2283 patients’ melanomas harbored a documented BRAF V600 mutation, and 1356 of these patients had documentation of systemic therapy for advanced disease at the clinical sites. Seven hundred and seven patients received front‐line treatment with a regimen other than BRAF/MEKi, aPD‐1, or niv/ipi, and 81 patients were excluded due to insufficient data or follow‐up. Two hundred and ninety seven were found to have received treatment with front‐line BRAF/MEKi (n = 35 vemurafenib/cobimetinib, n = 262 dabrafenib/trametinib), while 162 patients received treatment with front‐line aPD‐1 (n = 69 nivolumab, n = 93 pembrolizumab), and 108 patients received treatment with front‐line niv/ipi.

For the patients included in our analysis, the median patient was 62 years of age with an ECOG performance status of 1 and an LDH of 279 units/liter. Patients treated with front‐line aPD‐1 had a higher median age (66 vs 60 and 56 for BRAF/MEKi and niv/ipi, respectively), while patients treated with front‐line BRAF/MEKi had a higher median pretreatment LDH (271 vs 218 and 191 for niv/ipi and aPD‐1, respectively) and were more likely to have an elevated PS (32% vs 5% and 13% for niv/ipi and aPD‐1, respectively) (Table 1). There was no significant difference in the total number of systemic therapies that those in each treatment group received. At the time of analysis, patients treated with front‐line BRAF/MEKi were less likely to be alive at the time of analysis.

**Table 1:** Patient demographics

	Front‐line therapy			*P*‐value
	BRAF/MEKi	niv/ipi	aPD‐1	
Age				
N (Missing)	297 (0)	108 (0)	162 (0)	<.001^a^
Median (IQR)	60 (51‐69)	56 (49‐65)	66 (53‐77)	
N (Missing)	162 (135)	74 (34)	91 (71)	
LDH value				
Mean(SD)	507.69 (884)	311.77 (207)	293.01 (314)	.002^a^
Median(IQR)	271 (184‐492)	218 (172‐397)	191 (167‐309)	
N (Missing)	297 (0)	108 (0)	162 (0)	
Total Number of therapies received				
Mean(SD)	1.5 (0.79)	1.4 (0.82)	1.5 (0.75)	.180^a^
Median	1	1		
Range	1‐5	1‐5	1‐4	
Patient alive as last follow‐up				
No	160 (53.9%)	25 (23.2%)	52 (32.1%)	
Yes	137 (46.1%)	83 (76.9%)	110 (67.9%)	<.001^b^
Second‐line therapy received				
No	190 (64.0%)	78 (72.2%)	96 (59.3%)	
Yes	107 (36.0%)	30 (27.8%)	66 (40.7%)	.093^b^
aPD‐1				
N	45	0	0	
Niv/ipi				
N	21	0	4	
BRAF/MEKi				
N	8	30	33	
Other				
N	33	0	29	
Elevated ECOG				
ECOG >1	32 (10.8%)	5 (4.63%)	13 (8.02%)	
ECOG ≤1	137 (46.1%)	61 (56.5%)	99 (61.1%)	.014^b^
Missing ECOG	128 (43.1%)	42 (38.9%)	50 (30.9%)	

a Kruskal‐Wallis test.

b Chi‐square.

**Figure 1:**
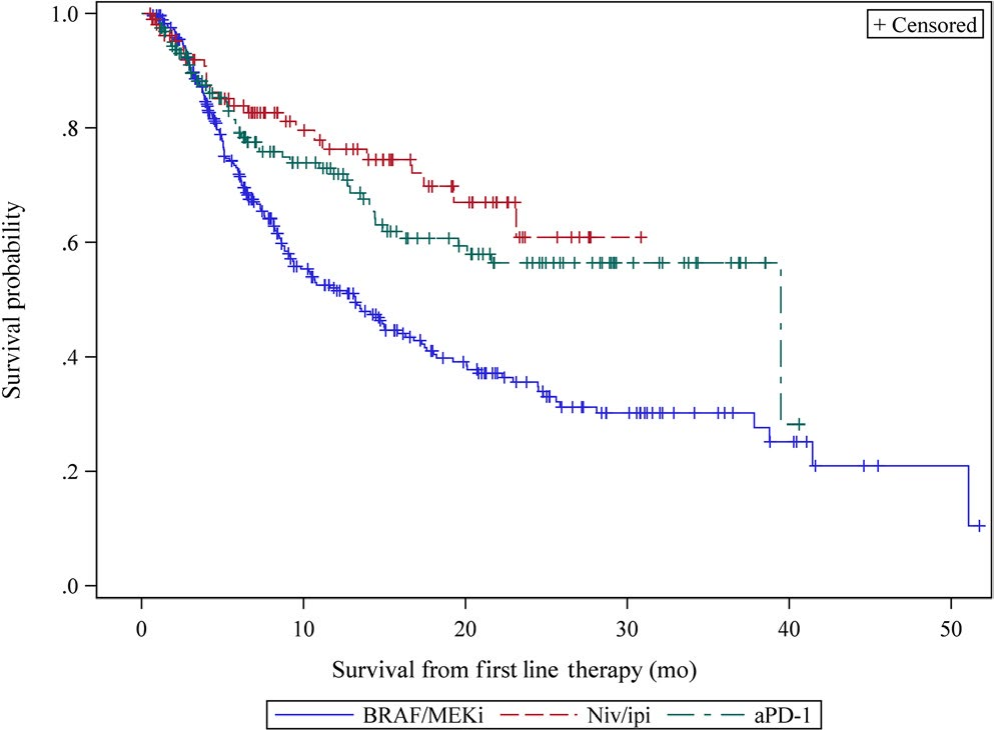
Overall survival of patients with advanced BRAF V600 Mutated Melanoma according to front‐line treatment

With a median follow up of 22.4 (interquartile range [IQR] 10.3‐32.7) months (m), the median OS for all treated patients was 19.3 m (IQR 6.2‐51.1 m). Median OS for patients treated with front‐line niv/ipi was not reached (NR) (IQR 8.7 – NR), while median OS for patients treated with front‐line aPD‐1 and BRAF/MEKi was 39.5 months (IQR 8.7‐NR) and 13.2 months (IQR 5.2‐41.4) (Figure 1). In univariate models of OS, front‐line treatment with niv/ipi (*P *≤ .001), age < 65 years (*P* = .007), ECOG 0‐1 (*P* ≤ .0001), and non‐elevated pretreatment LDH (*P* < .0001) were all associated with increased OS. Multivariate Cox regression analysis confirmed that these variables were associated with OS (Table 2). After adjustment for LDH, PS, and age, front‐line treatment with both aPD‐1 and niv/ipi were associated with prolonged OS compared to front‐line treatment with BRAF/MEKi. Although there was a trend towards prolonged OS for those treated with front‐line niv/ipi compared to aPD‐1 after adjusting for prognostic variables, the difference was not statistically significant (HR 0.77, 95%CI 0.47‐1.26).

**Table 2:** Multivariate cox regression of treatment and prognostic variables and effect on overall survival

	*P*‐value	Description	Hazard ratio	95% CI
Therapy	.0016	BRAF/MEKi vs niv/ipi	1.96	1.28‐3.01
		BRAF/MEKi vs aPD‐1	1.51	1.08‐2.1
		niv/ipi vs aPD‐1	0.77	0.47‐1.26
LDH	.0009	Elevated LDH vs Missing LDH	1.36	1.01‐1.84
		Elevated LDH vs Normal LDH	1.99	1.39‐2.85
		Missing LDH vs Normal LDH	1.46	1.05‐2.03
ECOG	.0021	ECOG >1 vs ECOG ≤1	2.08	1.38‐3.14
		ECOG >1 vs Missing ECOG	1.84	1.22‐2.78
		ECOG ≤1 vs Missing ECOG	0.88	0.67‐1.16
Age	.040	Age >64 vs Age ≤64	1.32	1.01‐1.71

**Figure 2:**
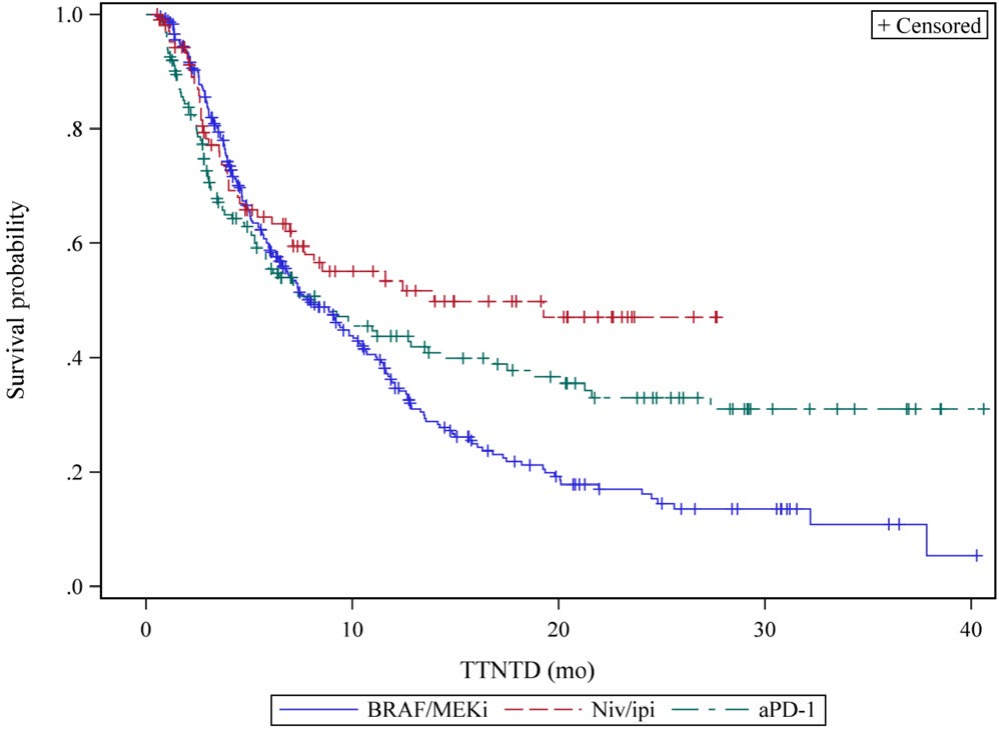
Time to next‐line therapy or death of patients for advanced BRAF V600 mutated melanomas by front‐line treatment

Median TTNTD for all treated patients was 8.6 months (IQR 3.6‐24.8). Median TTNTD of patients treated with front‐line niv/ipi was 14 months (IQR 3.6‐NR) while median TTNTD for patients treated with front‐line aPD‐1 and BRAF/MEKi was 8.6 months (IQR 2.8‐NR) and 8 months (IQR 3.9‐16.1), respectively (Figure 2. In univariate models of TTNTD, front‐line treatment with niv/ipi (*P* = .009), ECOG 0‐1 (*P* = .02), and normal pretreatment LDH (*P* = .0001) were associated with increased TTNTD. No difference in TTNTD was noted for patients according to age < 65 (*P* = .6). Multivariate Cox regression analyses confirmed front‐line treatment with niv/ipi and normal pretreatment LDH as statistically significant markers of TTNTD (Table 3). After adjusting for other prognostic factors, only elevated pretreatment LDH was associated with differences in TTNTD. However, there was a trend toward improved front‐line treatment with niv/ipi as compared to front‐line BRAF/MEKi, HR 1.48 95%CI 1.06‐2.07. No difference was noted in the TTNTD for patients treated with front‐line aPD‐1 as compared to BRAF/MEKi, HR 1.08 95%CI 0.83‐1.41.

**Table 3:** Multivariate cox regression of treatment and prognostic variables and effect on time to next therapy or death

	*P*‐value	Description	Hazard ratio	95% CI
Therapy	.071	BRAF/MEKi vs niv/ipi	1.48	1.06‐2.07
		BRAF/MEKi vs aPD‐1	1.08	0.83‐1.41
		niv/ipi vs aPD‐1	0.731	0.5‐1.06
LDH	.0041	Elevated LDH vs Missing LDH	1.31	1.01‐1.72
		Elevated LDH vs Normal LDH	1.65	1.23‐2.23
		Missing LDH vs Normal LDH	1.26	0.97‐1.63
ECOG	.093	ECOG >1 vs ECOG ≤1	1.42	0.98‐2.06
		ECOG >1 vs Missing ECOG	1.52	1.04‐2.22
		ECOG ≤1 vs Missing ECOG	1.07	0.85‐1.35
Age	.99	Age >64 vs Age ≤64	0.999	0.8‐1.25

In our sensitivity analysis, using a selected patient population with restriction of second‐line therapy, multivariate Cox regression confirmed the decreased likelihood of OS for patients treated with front‐line BRAF/MEKi as compared to those treated with front‐line niv/ipi (HR 2.11, 95%CI 1.34‐3.3) or aPD‐1 (HR 1.71, 95%CI 1.15‐2.55).

## 4 DISCUSSION

In our real‐world retrospective analysis, patients with advanced BRAF V600 mutated melanoma treated with front‐line niv/ipi or aPD‐1 had an increased likelihood of survival compared to those treated with front‐line BRAF/MEKi. This trend persisted after adjusting for known prognostic variables and was confirmed in both a cohort of patients who received any second‐line therapy and a cohort in which the second‐line therapy was restricted to the opposite or no therapy. Notably, these findings are in keeping with a recently reported retrospective review of 301 patients treated with either front‐line aPD‐1 or BRAF/MEKi.^9^

In this cohort of patients with metastatic BRAF V600 mutated melanoma, median OS for patients treated with front‐line niv/ipi or aPD‐1 was NR and 39.5 months. This is similar to the survival reported for patients with BRAF mutated melanoma treated with Checkmate 067.^10^ However, the median OS noted for patients in this study treated with front‐line BRAF/MEKi was significantly less, 13.2 months, than those treated on COMBI‐d, roughly 24 months.^11^ Our reported median OS for patients treated with front‐line BRAF/MEKi is similar to previously reported institutional experiences with front‐line dabrafenib and trametinib, 15.4 months.^12^ Additionally, the TTNTD for patients treated with front‐line BRAF/MEKi in our study, was similar to the PFS reported in another retrospective review of patients treated with BRAF/MEKi in the front‐line setting, 5.8 months.^13^ Of note, in this review, the authors note that 30.3% of patients treated with front‐line BRAF/MEKi had known brain metastasis and 48.5% of patients had an elevated LDH, which could suggest a selection bias of BRAK/MEKi for higher risk patients.

One possible explanation for the difference in our reported OS and that of COMBI‐D could be due to the presence of brain metastasis. Patients with active brain disease were excluded from both COMBI‐d and Checkmate 067. The presence of brain metastasis at the time of index treatment for patients included in this study using real‐world data is unknown. Therefore, the difference in OS for patients treated with the different front‐line therapies (aPD‐1, BRAF/MEKi, and niv/ipi) in this study could be due to an imbalance of patients with brain metastasis, or other unmeasured confounders, among the groups. However, the larger than expected difference in OS between patients treated with front‐line ICI and BRAF/MEKi could also be due to the differences in each of these treatment's effectiveness in real‐world patients who would not have met inclusion criteria the reported prospective trials. However, a recent trial suggests similar intracranial and extracranial efficacy of niv/ipi in patients with brain metastasis.^14^ Although intracranial response rates appear to be slightly less with aPD‐1 alone, as compared to extracranial response rates, responses have been shown to be long‐lasting.^15^ In contrast, a small phase 2 trial of dabrafenib and trametinib in patients with melanoma brain metastasis showed an initial high intracranial response rate (58%), however the median duration of response was 6.5 months, with few long‐term responses.^16^ Therefore, we postulate that a potential reason for the difference in OS between those treated with ICI and BRAF/MEKi in our study may be the inferior control of Central Nervous System disease of BRAF/MEKi compared to ICIs.

Interestingly, this study showed prolonged OS for patients treated with aPD‐1 compared to BRAF/MEKi, however, this difference was not due to a difference in TTNTD with each of these therapies. As TTNTD for the 75th percentile of patients treated with anti‐PD1 was not reached, it is possible that with longer follow‐up, a significant difference may be observed. However, it is also possible that the difference in OS seen between these two treated groups is not due to the treatments themselves, but rather the tumors’ resistance mechanisms to each treatment. It was previously shown that aPD‐1 therapy is markedly less effective for patients who had previously progressed on BRAF/MEKi.^7^ This has also been observed for BRAF/MEKi in patients who had previously progressed on aPD‐1 therapy, albeit to a lesser degree. Preclinical studies have shown cross resistance of BRAF/MEKi resistance mechanisms to immunotherapy, one of which is upregulation of AXL.^17^ In vivo models have shown that the expression of AXL reduces the effectiveness of aPD‐1 therapy. Inhibition of AXL has also been shown to sensitize tumors to aPD‐1 therapy.^18^

There are several potential limitations of this study. As described previously, data elements such as brain metastasis, symptomatic disease, overall disease burden, metastasectomy, radiation treatments, and disease burden are not readily available within the data. Therefore, we are unable to account for certain differences among patients which could have driven the selection of front‐line therapy. Although we attempted to control for a potential selection bias by using multivariate Cox regression with ECOG PS, age, and elevated baseline LDH, there was a significant proportion of patients for whom ECOG and LDH information was lacking. Given the prognostic importance of elevated ECOG and LDH, the latter of which has recently been correlated to roughly one half to two thirds the rate of OS at 5 years for patients treated with ICIs,^19^ it is likely that our attempt to correct for these differences may have been imperfect. However, we do note that the median OS for patients treated with front‐line BRAF/MEKi in our study is similar to reports of other institutional experiences,^12^ suggesting against a meaningful impact proportion of patients with poor prognosis.

## 5 CONCLUSIONS

In this real‐world, retrospective analysis, patients with advanced BRAF V600 mutant melanoma treated with front‐line immunotherapy had longer overall survival as compared to those treated with front‐line BRAF/MEKi. Validation of these findings is needed prior to being applied to clinical practice. These real‐world results may be validated upon completion and reporting of an ongoing randomized phase III trial, EA6134 which assesses the efficacy of front‐line niv/ipi followed by planned second‐line therapy of dabrafenib and trametinib as compared to the opposite treatment sequence.

## CONFLICT OF INTEREST

KFG has advised for BMS (within last year), Novartis (2 years ago), Array (within last year), Castle Biosciences (>3 years ago)—all less than 5k annually. SP received institutional research funding from Merck and Takeda. JRH is contracted with Ebix publishers to perform audio reviews of published articles in general surgery; this work is not related to melanoma. SL does consulting for Amgen, Merck, Genmab, Xencor, and BMS; receives research support from BMS, Merck, Vaccinex; and performs contracted research for Pfizer, Plexxikon, Genentech, Neon Therapeutics, Nektar, Astellas, F Star, Xencor.

## AUTHOR CONTRIBUTIONS

JM was involved in the conceptualization, data curation, formal analysis, and methodology and writing of the original draft. DC and JY were involved in formal analysis and writing‐reviewing and editing. SH, KG, SP, SC and JH were involved in the methodology, supervision, and writing‐review and editing.

## Data Availability

The data that support the findings of this study have been originated by Flatiron Health, Inc. These de‐identified data may be made available upon request, and are subject to a license agreement with Flatiron Health; interested researchers should contact http://www.DataAccess@flatiron.com to determine licensing terms

## References

[1] Brose MS , Volpe P , Feldman M , et al. BRAF and RAS mutations in human lung cancer and melanoma. Cancer Res. 2002;62(23):6997‐7000. 12460918

[2] Long GV , Stroyakovskiy D , Gogas H , et al. Combined BRAF and MEK inhibition versus BRAF inhibition alone in melanoma. N Engl J Med. 2014;371(20):1877‐1888. 25265492 10.1056/NEJMoa1406037

[3] Larkin J , Ascierto PA , Dréno B , et al. Combined vemurafenib and cobimetinib in BRAF‐mutated melanoma. N Engl J Med. 2014;371(20):1867‐1876. 25265494 10.1056/NEJMoa1408868

[4] Dummer R , Ascierto PA , Gogas HJ , et al. Encorafenib plus binimetinib versus vemurafenib or encorafenib in patients with BRAF‐mutant melanoma (COLUMBUS): a multicentre, open‐label, randomised phase 3 trial. Lancet Oncol. 2018;19(5):603‐615. 29573941 10.1016/S1470-2045(18)30142-6

[5] Long GV , Eroglu Z , Infante J , et al. Long‐term outcomes in patients with BRAF V600‐mutant metastatic melanoma who received dabrafenib combined with trametinib. J Clin Oncol. 2018;36(7):667‐673. 28991513 10.1200/JCO.2017.74.1025 PMC10466457

[6] Hodi FS , Chiarion‐Sileni V , Gonzalez R , et al. Nivolumab plus ipilimumab or nivolumab alone versus ipilimumab alone in advanced melanoma (CheckMate 067): 4‐year outcomes of a multicentre, randomised, phase 3 trial. Lancet Oncol. 2018;19(11):1480‐1492. 30361170 10.1016/S1470-2045(18)30700-9

[7] Johnson DB , Pectasides E , Feld E , et al. Sequencing treatment in BRAFV600 mutant melanoma: anti‐PD‐1 before and after BRAF inhibition. J Immunother. 2017;40(1):31‐35. 27846054 10.1097/CJI.0000000000000148 PMC10182887

[8] Curtis MD , Griffith SD , Tucker M , et al. Development and validation of a high‐quality composite real‐world mortality endpoint. Health Serv Res. 2018;53(6):4460‐4476. 29756355 10.1111/1475-6773.12872 PMC6232402

[9] Schilling B , Martens A , Geukes Foppen MH , et al. First‐line therapy‐stratified survival in BRAF‐mutant melanoma: a retrospective multicenter analysis. Cancer Immunol Immunother. 2019;68(5):765‐772. 30806748 10.1007/s00262-019-02311-1 PMC11028062

[10] Wolchok JD , Chiarion‐Sileni V , Gonzalez R , et al. Overall survival with combined nivolumab and ipilimumab in advanced melanoma. N Engl J Med. 2017;377(14):1345‐1356. 28889792 10.1056/NEJMoa1709684 PMC5706778

[11] Long GV , Flaherty KT , Stroyakovskiy D , et al. Dabrafenib plus trametinib versus dabrafenib monotherapy in patients with metastatic BRAF V600E/K‐mutant melanoma: long‐term survival and safety analysis of a phase 3 study. Ann Oncol. 2017;28(7):1631‐1639. 28475671 10.1093/annonc/mdx176 PMC5834102

[12] Cavalieri S , Di Guardo L , Cimminiello C , et al. Combined therapy with dabrafenib and trametinib in BRAF‐mutated metastatic melanoma in a real‐life setting: the INT Milan experience. Tumori. 2016;102(5):501‐507. 27470608 10.5301/tj.5000539

[13] Kreft S , Gesierich A , Eigentler T , et al. Efficacy of PD‐1‐based immunotherapy after radiologic progression on targeted therapy in stage IV melanoma. Eur J Cancer. 2019;116:207‐215. 31212163 10.1016/j.ejca.2019.05.015

[14] Tawbi HA , Forsyth PA , Algazi A , et al. Combined nivolumab and ipilimumab in melanoma metastatic to the brain. N Engl J Med. 2018;379(8):722‐730. 30134131 10.1056/NEJMoa1805453 PMC8011001

[15] Goldberg SB , Gettinger SN , Mahajan A , et al. Pembrolizumab for patients with melanoma or non‐small‐cell lung cancer and untreated brain metastases: early analysis of a non‐randomised, open‐label, phase 2 trial. Lancet Oncol. 2016;17(7):976‐983. 27267608 10.1016/S1470-2045(16)30053-5 PMC5526047

[16] Davies MA , Saiag P , Robert C , et al. Dabrafenib plus trametinib in patients with BRAF. Lancet Oncol. 2017;18(7):863‐873. 28592387 10.1016/S1470-2045(17)30429-1 PMC5991615

[17] Müller J , Krijgsman O , Tsoi J , et al. Low MITF/AXL ratio predicts early resistance to multiple targeted drugs in melanoma. Nat Commun. 2014;5:5712. 25502142 10.1038/ncomms6712 PMC4428333

[18] Guo Z , Li Y , Zhang D , Ma J . Axl inhibition induces the antitumor immune response which can be further potentiated by PD‐1 blockade in the mouse cancer models. Oncotarget. 2017;8(52):89761‐89774. 29163786 10.18632/oncotarget.21125 PMC5685707

[19] Larkin J , Chiarion‐Sileni V , Gonzalez R , et al. Five‐year survival with combined nivolumab and ipilimumab in advanced melanoma. N Engl J Med. 2019;381(16):1535‐1546. 31562797 10.1056/NEJMoa1910836

